# Comment on ‘A Clinically Aligned Two‐Stage Machine Learning Framework for Predicting Hungry Bone Syndrome After Parathyroidectomy’

**DOI:** 10.1002/edm2.70310

**Published:** 2026-08-12

**Authors:** Aman Batool Hashmi, Maliha Khalil, Syeda Bareera

**Affiliations:** ^1^ Khyber Girls Medical College Peshawar Pakistan; ^2^ Khyber Medical University Peshawar Pakistan

To the Editor,

1

We read with great interest the recent article by Yin et al. [1] Strengths include the size of the cohort, using training data to fit preprocessing parameters and models, and reporting discrimination and calibration measures. The proposed workflow also mirrors the perioperative sequence in which the information is gathered for the preoperative stage, followed by the findings of the intraoperative stage. However, there are three concerns about the incremental predictive value and suggested clinical utility of the framework.

The main concern is that the study does not clearly show what the two‐stage design adds. Within this framework, EasyEnsemble achieved an AUROC of 0.776, which was very close to the 0.772 reported for logistic regression [1]. The single‐stage and no‐SMOTE models produced slightly higher AUROCs of 0.782 and 0.786, although their recall was lower at the selected thresholds [1]. Overall, these results point to a trade‐off: the chosen model achieved better sensitivity, whereas the alternative models showed slightly stronger overall discrimination. Therefore, the results do not clearly establish that the two‐stage EasyEnsemble model is superior. The authors performed an ablation analysis of single‐stage and preoperative‐only variants, but did not present formal statistical comparisons of the change in discrimination or calibration, nor did they report reclassification or clinical net‐benefit analyses. Machine‐learning techniques do not always outperform well‐established logistic models [2], so the complexity should be warranted by the incremental predictive or clinical benefits. To enable proper appraisal, it is also important to report the development of the model, comparative evaluation, and intended clinical use transparently, following TRIPOD+AI guidance [3].

The proposed low‐, moderate‐, and high‐risk categories also require caution. The low‐risk group contained only 11 of the 177 test patients, and two still developed HBS, producing an observed rate of 18.2% [1]. Confidence intervals for these threshold‐specific estimates were not reported. Although the authors explain the sensitivity‐ and specificity‐oriented rationale for the 35% and 85% thresholds, they do not state whether these thresholds were prespecified or selected within the training data before evaluation in the test set. More importantly, sensitivity, specificity, and predictive values do not establish whether threshold‐guided management would improve decisions or outcomes compared with the existing protocol of routine calcium and calcitriol supplementation, biochemical monitoring, and clinically indicated treatment escalation. Decision‐curve analysis is specifically intended to evaluate such net clinical benefit [4].

Lastly, random splitting of patients from a 17‐year period into training and test sets is only an internal validation. Patients treated at the same Centre by the same group of surgeons during the same time periods and using similar dialysis and supplementation regimens could then be split between the two datasets. This approach can maintain patterns of performance that are Centre‐specific and era‐specific but not test performance in actual future patients. The model should first be tested in patients treated later at the same Centre to determine whether its performance remains stable as clinical practice changes. Independent external validation is also needed to assess whether the model performs reliably in other populations and healthcare settings [5].

In conclusion, this two‐stage framework represents a promising approach to HBS risk prediction. Future research should include temporal validation and independent multicenter external validation, compare the framework with simpler models with the same predictors, the same outcome definitions and the same evaluation data, and validate the clinical utility of the proposed risk thresholds. Prospective evaluation should then follow to see if model‐guided care enhances clinical decision making and patient outcomes. Such evidence would increase confidence in the model and help determine whether it should be used to guide individualized postoperative care.

## Author Contributions

**Aman Batool Hashmi:** conceptualization, investigation, writing – original draft, methodology, validation, visualization, writing – review and editing, software, formal analysis, project administration, resources, supervision. **Syeda Bareera:** writing – review and editing. **Maliha Khalil:** visualization.

## Funding

The authors have nothing to report.

## Ethics Statement

The authors have nothing to report.

## Consent

The authors have nothing to report.

## Conflicts of Interest

The authors declare no conflicts of interest.

## Data Availability

No new data were generated in this work.
